# Hybrid incompatibilities are affected by dominance and dosage in the haplodiploid wasp *Nasonia*

**DOI:** 10.3389/fgene.2015.00140

**Published:** 2015-04-15

**Authors:** Leo W. Beukeboom, Tosca Koevoets, Hernán E. Morales, Steven Ferber, Louis van de Zande

**Affiliations:** ^1^Evolutionary Genetics, Groningen Institute for Evolutionary Life Sciences, University of GroningenGroningen, Netherlands; ^2^School of Biological Sciences, Monash UniversityMelbourne, VIC, Australia

**Keywords:** sex, ploidy, hybrid, cytonuclear incompatibility, haplodiploidy, dominance, dosage, Haldane's rule

## Abstract

Study of genome incompatibilities in species hybrids is important for understanding the genetic basis of reproductive isolation and speciation. According to Haldane's rule hybridization affects the heterogametic sex more than the homogametic sex. Several theories have been proposed that attribute asymmetry in hybridization effects to either phenotype (sex) or genotype (heterogamety). Here we investigate the genetic basis of hybrid genome incompatibility in the haplodiploid wasp *Nasonia* using the powerful features of haploid males and sex reversal. We separately investigate the effects of heterozygosity (ploidy level) and sex by generating sex reversed diploid hybrid males and comparing them to genotypically similar haploid hybrid males and diploid hybrid females. Hybrid effects of sterility were more pronounced than of inviability, and were particularly strong in haploid males, but weak to absent in diploid males and females, indicating a strong ploidy level but no sex specific effect. Molecular markers identified a number of genomic regions associated with hybrid inviability in haploid males that disappeared under diploidy in both hybrid males and females. Hybrid inviability was rescued by dominance effects at some genomic regions, but aggravated or alleviated by dosage effects at other regions, consistent with cytonuclear incompatibilities. Dosage effects underlying Bateson–Dobzhansky–Muller (BDM) incompatibilities need more consideration in explaining Haldane's rule in diploid systems.

## Introduction

The combination of genomes from two species into hybrid progeny often leads to inviability, sterility, and other negative fitness effects, a phenomenon known as hybrid incompatibility. Hybrid incompatibility may also be manifested when individuals from different populations, that have been separated for a long time, meet and reproduce. Such post-zygotic reproductive barriers between individuals of diverging populations are an important driving force of speciation (Coyne and Orr, 2004). Negative epistatic gene-interactions are considered as the genetic cause for incompatibilities between diverged genomes. They were first described by Bateson (1909), Dobzhansky (1937), and Muller (1942), and are therefore termed Bateson–Dobzhansky–Muller (BDM) incompatibilities (Lowry et al., 2008; Presgraves, 2010).

A well-known pattern during the initial stages of speciation, involving sexual reproduction, was described by Haldane (1922): “When in the F_1_ offspring of two different animal races one sex is absent, rare or sterile, that sex is the heterozygous [heterogametic] sex.” This observation has fueled a large number of studies into the genetic basis of hybrid genome incompatibilities. Theories explaining Haldane's rule are based on the assumption that co-adapted gene-complexes become disrupted in hybrid genomes, and that sex chromosomes play a disproportional role in this interaction.

Some of the main theories that are discerned as the cause of Haldane's rule are the dominance, faster-male, and faster-X theories. The dominance theory assumes that deleterious mutations are (partially) recessive and thus masked by heterozygosity (Turelli and Orr, 1995). Negative autosomal interactions are often not expressed due to positive interactions that are dominant in diploids, irrespective of sex. However, negative interactions involving genes located on sex chromosomes are not rescued by dominance in the heterogametic sex, resulting in a higher chance to express hybrid incompatibilities. Supporting evidence for the dominance theory comes from laborious introgression studies that show a major effect of dominance in hybridizations that follow Haldane's rule (Turelli and Orr, 1995; True et al., 1996; Jiggins et al., 2001; Presgraves, 2003; Tao and Hartl, 2003; Slotman et al., 2005; Bierne et al., 2006; Schilthuizen et al., 2011).

The faster-male theory states that, due to stronger sexual selection on males through female choice and male–male competition, male-specific genes evolve faster than female-specific genes. In support of this theory, genes that are male-biased in their expression show greater divergence between species compared to female-biased and non-biased genes (Civetta and Singh, 1995; Meiklejohn et al., 2003; Hearty et al., 2007, but see Metta et al., 2006 for a counter example). In addition, spermatogenesis is considered to be more easily disrupted by mutations than oogenesis, leading to more male than female hybrid sterility (Wu and Davis, 1993). Indeed, diverged loci causing male sterility are far more numerous than loci causing female sterility, whereas lethal incompatibilities are equally frequent in males and females (Hollocher and Wu, 1996; True et al., 1996; Tao et al., 2003; Mishra and Singh, 2005). The faster-male theory only applies to species in which males are the heterogametic sex (under female heterogamety it predicts that males suffer most from hybridization, but the opposite is observed), and this theory can therefore only partly explain Haldane's rule.

The faster-X theory predicts an overall faster evolution of sex chromosomes than autosomes, due to more efficient selection of recessive beneficial mutations under haploidy (Charlesworth et al., 1987). This makes hemizygous sex chromosomes more prone to be involved in disrupted gene-interactions in hybrid genomes than the more slowly evolving autosomes. Both supporting (Ford and Aquadro, 1996; Begun et al., 2007; Baines et al., 2008) and contradicting (Betancourt et al., 2002; Thornton et al., 2006; Mank et al., 2010) results have been found for the faster-X theory. Moreover, the faster-X theory only explains the involvement of the sex chromosomes in hybrid incompatibilities, but additionally relies on dominance effects to explain Haldane's rule.

Although Haldane's rule seems to suggest that differences in hybridization effects between the sexes are due to differences in sex chromosome composition, hybrid incompatibilities do not only occur between autosomes and sex chromosomes. They may also occur between genes located in the nuclear genome and in the cytoplasm, such as chloroplast and mitochondrial genes, called cytonuclear incompatibilities (reviewed in Burton et al., 2006). Following studies by Gadau et al. (1999), Niehuis et al. (2008), and Ellison et al. (2008); Koevoets and Beukeboom (2009) argued that haplodiploid reproductive systems in which chromosomes that occur in haploidy in the male sex and in diploidy in the female sex might prove useful for studies of negative epistatic gene-interactions that cause hybrid incompatibilities.

Under haplodiploidy, females develop from fertilized eggs and are diploid, whereas males develop from unfertilized eggs and are haploid. As a consequence, an interspecific cross yields diploid hybrid F_1_ females with a maternal and paternal genome set, and haploid pure-species F_1_ males with only a maternal genome set. The first generation of recombinant hybrid males is produced by the hybrid F_1_ females. Although this is usually considered the F_2_ generation, it is technically the F_1_ hybrid male generation. Under haplodiploidy BDM can occur between all autosomal pairs and are not restricted to autosomes and the X chromosome as in diploids (Koevoets and Beukeboom, 2009). Dominance, faster-male and faster-X effects are all expected to apply to haplodiploids because the whole genome can be considered to be inherited as if it were an X chromosome in haploid males. Dominance effects in cytonuclear incompatibilities may be revealed by comparison of haploid and diploid hybrids. However, despite the fact that Haldane himself clearly included haplodiploids in the definition of his rule, studies into the genetic basis of hybrid incompatibilities have almost exclusively focused on diploid organisms with specialized sex chromosomes (Koevoets and Beukeboom, 2009; Schilthuizen et al., 2011).

A problem with almost all studies into the genetic basis of hybrid incompatibilities is that effects of sex and ploidy level could not be independently manipulated, because haploid or polyploid individuals in diploid species are typically lethal. A unique exception is the study of Malone and Michalak (2008) who measured hybrid sterility in sex reversed *Xenopus* frogs that have female heterogamety. Both normal (ZZ) and sex reversed (ZW) hybrid males were sterile, whereas both types of females were fertile. This shows that it is the phenotypic sex rather than the genotype that is responsible for the observed sex differences in severity of incompatibilities, consistent with the faster male theory. Additional studies are clearly needed that are discriminative between theories by experimental manipulation of genotype and sex in a broader of range of organisms.

The haplodiploid genus *Nasonia* offers unique opportunities for investigating the genetic basis of hybrid incompatibilities. *Nasonia* species are reproductively isolated in nature due to species-specific infections with *Wolbachia* bacteria that cause cytoplasmic incompatibility in interspecies crosses (Breeuwer and Werren, 1990, 1995; Bordenstein et al., 2001). Antibiotic treatment of laboratory strains allows one to set up interspecific crosses and to assess post-zygotic isolation due to genome divergence. Such studies have revealed the involvement of specific genomic regions in hybrid breakdown and an important role for disrupted interactions between nuclear and cytoplasmic genes (Gadau et al., 1999; Niehuis et al., 2008; Koevoets et al., 2012a). Although *Nasonia* males are normally haploid, mutant strains that consist of triploid females and diploid males have been studied for over 60 years (Whiting, 1960). Diploid males are fully fertile, produce diploid sperm and father triploid female offspring (Beukeboom and Kamping, 2006). Recent progress in research into the mechanism of sex determination in *Nasonia* (Verhulst et al., 2010) showed that prevention of maternal input of *transformer* (*tra*) mRNA into fertilized eggs by RNA interference, leads to development of diploid males rather than females. This makes it possible to generate diploid individuals that are genetically identical to diploid females, but are phenotypically functional males.

It has previously been shown for several *Nasonia* species pairs that haploid hybrid males suffer more from incompatibilities than diploid hybrid females (Niehuis et al., 2008; Koevoets et al., 2012a). A problem with these studies is that haploid F_2_ hybrid males resulted from recombination in F_1_ hybrid females that carried one intact chromosomal set of each parental species. This prevented a good comparison of hybrid effects between males and females of similar genetic composition. Here we address whether haploid hybrid males of *Nasonia vitripennis* and *Nasonia longicornis* suffer more from cytonuclear incompatibilities due to their genotype (ploidy) or phenotype (sex). In order to do so, we generate diploid hybrid males with one chromosomal set of each parental species and compare their sterility and inviability levels to diploid hybrid females of similar genetic make-up, as well as to haploid hybrid males. We do the same for diploid hybrid males and females that are on average 50% homozygous for one species genome and hybrid for the remainder of the genome. By doing these crosses in two directions, we can also test for effects of different cytoplasm. We further screen hybrid nuclear genomes with 32 microsatellite markers that are distributed across the entire genome to identify genomic regions associated with hybrid inviability. The results shed light on the genetic causes of hybrid incompatibilities and provide evidence for theories that explain Haldane's rule.

## Materials and methods

### Strains and crosses

**Figure 1:**
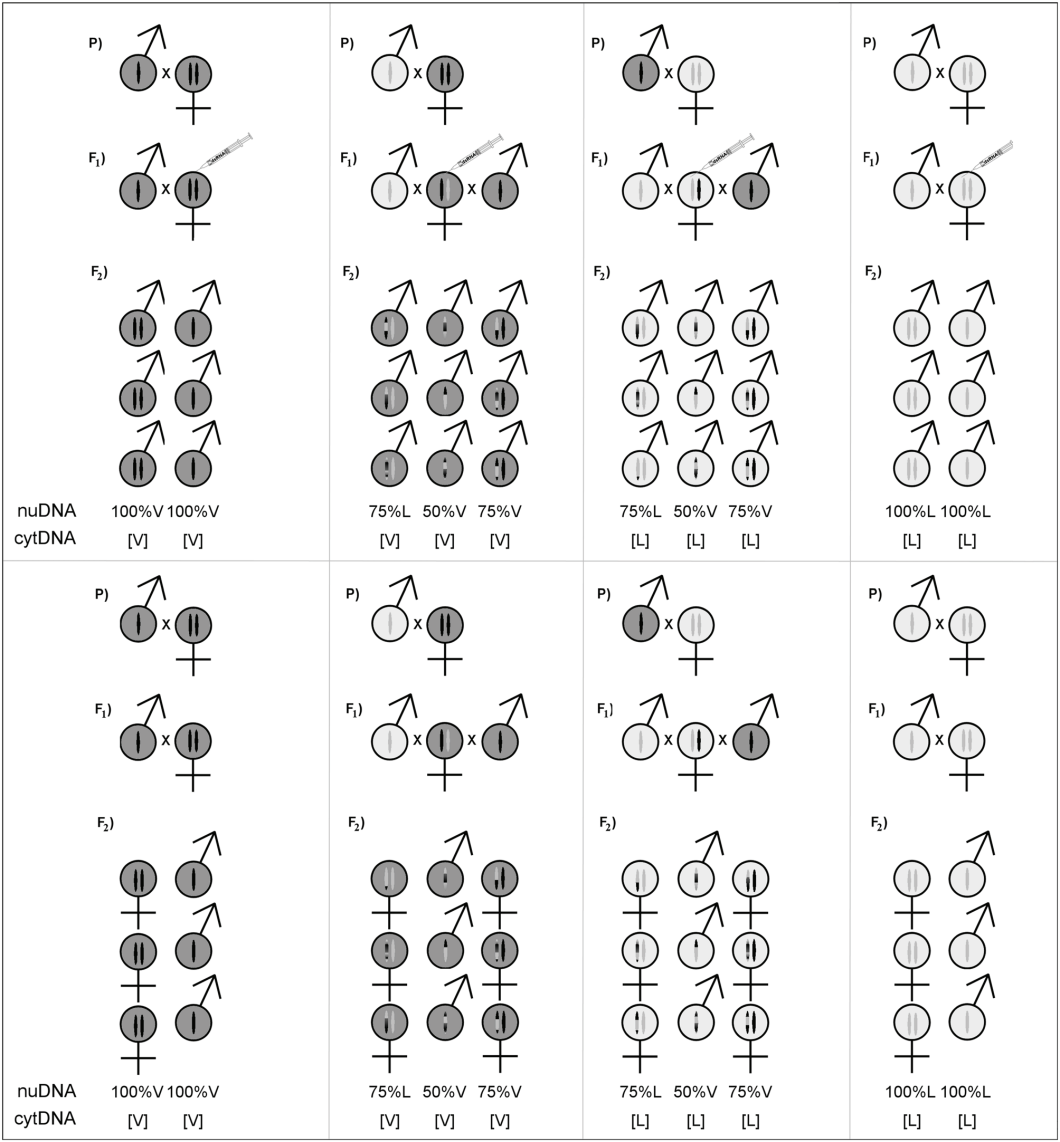
**Cross design for testing sex- and genotype effects on hybrid incompatibilities**. Left and right panels show the pure species crosses, the middle panes show the reciprocal hybrid crosses with double backcross design. The top panes show the crosses treated with RNAi against *transformer* to produce diploid male offspring, the bottom panes show the control crosses with regular diploid female offspring. In all cases, the haploid offspring are male. The average genetic composition of the offspring is indicated as the percentage of nuclear genome (nuDNA) of one species (the remaining part is of the other species) and cytotype (cytDNA) between square brackets. The genetic composition of haploids is the recombined maternal composition; a random mix of 50% of alleles of either species. The genetic composition of diploids is the recombined maternal genome plus a complete paternal genome.

Wolbachia-free *N. vitripennis* (AsymC) and *N. longicornis* (IV7R2) strains were used, reared on *Calliphora* sp. fly pupae, at 25°C under constant light. F_1_ hybrid females were created by crossing *N. vitripennis* and *N. longicornis* males and females reciprocally. These hybrid females carry 50% of the genome of either species and are referred to as VL[L] and LV[V] with the letter between brackets indicating the origin of the maternally inherited cytotype. Hybrid females were backcrossed 48 h after eclosion to virgin males of either species according to the scheme shown in Figure 1. This yielded haploid hybrid males from unfertilized eggs with 50% of the genome of either species as well as diploid hybrid females with 75% of the genome of the backcross species and 25% of the other species.

To generate hybrid diploid males, F_1_ hybrid female pupae were collected ±8 days after oviposition and injected with double-stranded RNA against *transformer* based on either *N. vitripennis* or *N. longicornis* RNA according to the cytotype of the females (see Lynch and Desplan, 2006 and Verhulst et al., 2010 for details of the parental RNAi treatment). Untreated pupae were left to continue development and served as control for the viability of the sex reversed males. Before being used in experiments, females were collected 1–2 days prior to eclosion and provided with hosts for feeding and ovarian development.

Two types of F_2_ haploid males were obtained from unfertilized eggs of F_1_ hybrid females (V and L cytotypes, each with 50% V and 50% L nuclear genome) (Figure 1). Backcrossing diploid hybrid F_1_ females with males of either parental species resulted in four classes of hybrid F_2_ female offspring with V and L cytotypes and largely matching or mismatching nuclear genome (i.e., 75% V and 25% L or 75% L and 25% V). For example, diploid individuals with 75% vitripennis genome and 25% longicornis genome are on average 50% homozygous for the vitripennis nuclear genome and for 50% hybrid for the vitripennis and longicornis nuclear genome. Such hybrid individuals are referred to as 75% V[V] and 75% V[L] for the vitripennis and longicornis cytotype, respectively. RNAi treatment of F_1_ hybrid females yielded genomically similar diploid hybrid males of each class. As controls served pure species individuals, i.e., 100% V[V] and 100% L[L] haploid and diploid males as well as diploid females.

### Measurement of hybrid incompatibilities

Hybrid incompatibility levels of F_2_ haploid males and diploid males and females were measured as (1) egg-to-adult survival: the number of oviposited eggs that developed into adult wasps, (2) behavioral sterility: proportion of males that proceed to full copulation upon being presented with a female, (3) physiological sterility: for males, no sired offspring following copulation, termed spermatogenic failure; for females, number of viable offspring (fecundity), and number of female offspring (fertilization ability), and (4) overall sterility: combining both sterility measures into the fraction of wasps that did not reproduce.

#### Egg-to-adult survival

F_1_ females were provided with two hosts for oviposition every day. One parasitized fly pupa was then stored in Carnoy's fixative at −20°C and the other placed at 25°C for wasp development for about 11 days following oviposition. Fixated hosts were opened to count the number of oviposited eggs. Hosts placed at 25°C were opened prior to wasp eclosion to count the number of developing wasps. As hybridization disrupts developmental rate in *Nasonia* to some extent, various developmental stages are encountered among hybrid offspring after 11 days of development, while all pure species offspring are typically in the same late instar pupal stage. Three categories were distinguished to quantify developmental disruptions: underdeveloped wasps (larvae and young pupae), diapause larvae and late instar wasp pupae. Late instar wasp pupae of control crosses were classified into female (diploid) or male (haploid) based on visual differences in morphology (wing size, presence of ovipositor). In case of all-male progenies from RNAi treated mothers, that consist of both haploid males from unfertilized eggs and diploid males from fertilized eggs, ploidy level was determined with flow cytometry for pure species progenies following the protocol described by de Boer et al. (2007), and with microsatellites for hybrid progenies. Data were cleared for unsuccessful matings resulting in all-male haploid progenies and for unsuccessful RNAi treatment as identified by clutches containing female offspring. Clutches containing only diapause larvae were also discarded, as successful mating and RNAi treatment could not be validated. RNAi treatment resulted in more all-diapause progenies which is likely the result of a general stress response. Mixed clutches of both diapause larvae and non-diapause individuals were included in the analysis as hybrid breakdown may also be manifested as an increase in production of diapause offspring. Viability of diapause larvae was confirmed by their continued development into adult individuals after placement at 25°C following a cold period (4°C) of several weeks.

Survival rates were determined by comparing the average number of oviposited eggs from the fixated hosts to the average number of viable offspring from the hosts in which the wasps were allowed to develop for 11 days. Ninety-five percentage confidence limits were used to compare the survival rates of experimental and control clutches.

#### Sterility measures

F_2_ offspring from clutches that were allowed to develop to adulthood were screened for behavioral and physiological sterility. Males were isolated upon eclosion and provided with a virgin *N. vitripennis* female for 10 min at 25°C. Only pure *N. longicornis* males were tested with *N. longicornis* females to avoid possible effects of mate discrimination (*N. vitripennis* females are less willing to mate with *N. longicornis* males, Giesbers et al., 2013). Male courtship behavior was observed and scored into seven successive categories: “no interest,” “interest,” “mounting” (positioning on top of the female), “display” (ritualized courtship display), “attempted copulation”(<5 s), “incomplete copulation” (12–14 s, no post-copulatory display), and “complete copulation” (12–14 s, including post-copulatory display) (modified after Clark et al., 2010). Males are either categorized as behaviorally sterile, meaning that they interrupted the courtship process during one of the first six categories, or as behaviorally fertile, meaning that they proceeded to complete copulation.

After the observations, males were stored at −20°C, unmated females were removed and mated females were isolated for 24 h and then provided with three hosts for 48 h to check their emerging offspring for the presence of (F_3_) daughters. The absence of female offspring following copulation is an indirect measure of male infertility and is interpreted as “male spermatogenic failure.” The levels of behavioral sterility (behaviors that do not lead to successful copulation), spermatogenic sterility (no female offspring after observed mating), and overall sterility (no female offspring out of total sample size) were compared between haploid and diploid, pure and hybrid, males with χ^2^-tests on proportions. A Tukey-type multiple comparison test was performed to pairwise test effects of genetic compositions of the hybrids (Zar, 1999) with Bonferroni correction for multiple testing.

Females were isolated upon eclosion and provided with a virgin male for 10 min at 25°C. Pure species females were tested with a conspecific male; hybrid females were tested with either *N. vitripennis* or *N. longicornis* males randomly. Female courtship behavior was scored in five successive categories: “no interest,” “interest,” “mounted” (arrests and allows the male to mount), “attempted copulation” (<5 s) and “copulation” (12–14 s). Female were scored as behaviorally sterile if they interrupted the mating sequence in one of the first four stages, and as behaviorally fertile if they proceeded to (complete) copulation. After the behavioral observations, all mated and unmated females were isolated for 24 h and provided with three hosts for 48 h to assess their fecundity (ability to produce viable offspring). Mated females were also tested for their egg fertilization ability by scoring the production of (F_3_) daughters.

The levels of behavioral sterility (behaviors that do not lead to successful copulation), fecundity (viable offspring), fertilization ability (female offspring) and overall sterility (number of clutches without female offspring out of total sample size) were compared between the groups with χ^2^-tests on proportions. A Tukey-type multiple comparison test was performed to pairwise test effects of genetic compositions of the hybrids (Zar, 1999) with Bonferroni correction for multiple testing.

### Transmission ratio distortion

Thirty-two microsatellite markers were previously developed from the genome sequences of an inbred line of *N. vitripennis* (V) and *N. longicornis* (L) (Werren et al., 2010; Koevoets et al., 2012a). They represent all sections of the genome of these homogeneous lines that were also used for this study. The transmission of V and L alleles was tested for deviation from the expected 1:1 ratio among offspring with χ^2^-tests after Yates and sequential Bonferroni corrections. The genomic regions that deviated significantly from equality are referred to as distorted regions and are potentially linked to transmission ratio distortion loci (or TRDLs) associated with hybrid inviability (Niehuis et al., 2008). All types of hybrid males (diploid 75%V/25%L[V], 25%V/75%L[V], 75%V/25%L[L], 25%V/75%L[L] and haploid 50%V/50%L[V] and 50%V/50%L[L]) were genotyped, as well as diploid hybrid females (25%V/75%L[V] and 75%V/25%L[L]) that had a partial mismatch between the nuclear and cytoplasmic genome.

Wasp DNA was extracted using either the filter-plate based method (Koevoets et al., 2012a) or the high salt-chloroform protocol (Maniatis et al., 1982). Microsatellite markers were amplified using the Qiagen multiplex PCR kit according to the manufacturer's recommendations (PCR profile: 15 min at 95°C, followed by 30 cycles of 30 s at 94°C, 1.5 min at TA and 1 min at 72°C, followed by 45 min at 72°C). DNA was amplified in 5 μL volumes using Applied Biosystems Veriti or Applied Biosystems 9700 thermocyclers. PCR products were diluted 400 times, separated on the Applied Biosystems 3730 DNA Analyzer and analyzed with GeneMapper v4.0 (Applied Biosystems). All fragment chromatograms were compared to those of adjacent markers to reveal genotyping errors or data inconsistencies. Only markers Nv121 and Nv307 in the 75%V/25%L[V] males yielded low genotyping confidence and the recovery biases of these markers are likely erroneous and cannot be confidently associated with hybrid inviability.

Regression analyses were performed to identify correlations between allelic recovery rates of microsatellites to identify genomic regions involved in cytonuclear incompatibilities across cross-types, sexes and ploidy levels. For every pairwise comparison markers were ordered based on the allelic recovery (*p*-value of χ^2^-test) and correlation estimated with regression analyses in SigmaPlot 11.0.1. This method can indicate genomic regions involved in inducing inviability that are not identified using the χ^2^-tests on the 1:1 ratio of alleles, as Yates and sequential Bonferroni corrections may reduce the likelihood of discovering such regions.

## Results

### Hybrid inviability

Hybrid survival was indirectly measured by comparing clutch size in one of two hosts with brood size of the second host within each experimental group. Mortality rates may be somewhat underestimated as the data suggest that not always all eggs were found. This is evident from somewhat higher survival rates than 100 percent in a few groups (Table 1). Control haploid and diploid males and females had survival rates of 92% or higher. Haploid hybrid males have significantly (based on non-overlapping 95% confidence limits) higher mortality rates than pure species males, up to 25% in F_2_ hybrid males with longicornis [L] cytotype. For diploid males and females survival rates are harder to measure because they derive from fertilized eggs and are always in clutches together with haploid males, as mated females typically lay a small proportion of unfertilized eggs, and eggs cannot be sexed. This means that comparison of clutch sizes with brood sizes is only possible between experimental groups, i.e., crosses in which diploid male hybrids are known to be produced can be compared to crosses that yield diploid hybrid females. In addition, these mixed haploid and diploid brood survival rates can be compared to haploid broods of virgin hybrid females. The results show that survival rates vary from 80 to 100% depending on cross type. Diploid hybrid females with vitripennis [V] cytotype have 100% survival but with longicornis [L] cytotype show some lethality (78–85% survival). Diploid hybrid males also suffered some lethality (80–95% survival) compared to diploid pure species males, except for 75% L[L] hybrids, which have survival rates with a 95% confidence range that overlap with the controls. Overall, survival rates of the four categories of diploid hybrid males overlapped with those of diploid hybrid females (Table 1). Assuming that the proportion of diploid individuals in broods with diploid males is similar to broods with diploid females, these results show that diploid hybrid males have similar survival as haploid hybrid males and diploid hybrid females.

**Table 1:** **Survival rates of haploid and diploid males and females from pure species and hybrid crosses**.

**Cross type**	**Parental cross**	**Progeny types**	**Focus individuals**	**Number of clutches eggs/adults**	**Average survival [95% confidence]**
Control	Diploid V[V] virgin ♀	Haploid V[V]♂	Haploid control ♂	24/25	92.7 [±7.2]
Control	Diploid L[L] virgin ♀	Haploid L[L]♂	Haploid control ♂	25/26	115.3 [±9.0]
Hybrid	Diploid LV[V] virgin ♀	Haploid L/V[V] ♂	Haploid hybrid ♂	43/43	78.2 [±6.0]
Hybrid	Diploid VL[L] virgin ♀	Haploid V/L[L] ♂	Haploid hybrid ♂	20/19	75.3 [±6.6]
Control	Haploid V[V] ♂ × diploid V[V] ♀	Haploid V[V] ♂ and diploid V[V] ♀	Diploid control ♂	35/34	100 [±4.6]
Control	Haploid L[L] ♂ × diploid L[L] ♀	Haploid L[L] ♂ and diploid L[L] ♀	Diploid control ♀	24/18	92 [±4.1]
Hybrid	Haploid V[V] ♂ × diploid LV[V] ♀	Haploid L/V[V] ♂ and diploid VV/VL[V]♂	Diploid hybrid ♀	33/29	95 [±5.0]
Hybrid	Haploid L[L] ♂ × diploid LV[V] ♀	Haploid L/V[V] ♂ and diploid VL/LL[V]♀	Diploid hybrid ♀	10/10	115 [±2.3]
Hybrid	Haploid V[V] ♂ × diploid VL[L] ♀	Haploid V/L[V] ♂ and diploid VV/VL[L] ♀	Diploid hybrid ♀	31/26	85 [±4.5]
Hybrid	Haploid L[L] ♂ × diploid VL[L] ♀	Haploid V/L[V] ♂ and diploid VL/LL[L] ♀	Diploid hybrid ♀	17/15	78 [±4.1]
Control	Haploid V[V] ♂ × diploid injected V[V] ♀	Haploid V[V] ♂ and diploid V[V] ♂	Diploid control ♂	7/5	113 [±7.5]
Control	Haploid L[L] ♂ × diploid injected L[L] ♀	Haploid L[L] ♂ and diploid L[L] ♂	Diploid control ♂	13/12	104 [±6.9]
Hybrid	Haploid V[V] ♂ × diploid injected LV[V] ♀	Haploid L/V[V] ♂ and diploid VV/VL[V] ♂	Diploid hybrid ♂	29/16	80 [±6.0]
Hybrid	Haploid L[L] ♂ × diploid injected LV[V] ♀	Haploid L/V[V] ♂ and diploid VL/LL[V] ♂	Diploid hybrid ♂	22/14	78 [±5.1]
Hybrid	Haploid V[V] ♂ × diploid injected VL[L] ♀	Haploid V/L[V] ♂ and diploid VV/VL[L] ♂	Diploid hybrid ♂	24/18	80 [±4.2]
Hybrid	Haploid L[L] ♂ × diploid injected VL[L] ♀	Haploid V/L[V] ♂ and diploid VL/LL[L] ♂	Diploid hybrid ♂	19/19	95[±4.5]

*Progeny types are indicated as the nuclear allelic combinations that can occur, i.e., in haploids either one L or one V allele, in diploids either a homospecific (VV or LL) or a heterospecific (VL) combination. Survival rate is determined by comparing clutch size in one of two hosts with brood size in the other host after development of eggs to adulthood. V, vitripennis, L, longicornis, letter in square brackets indicates species cytotype*.

### Hybrid sterility

#### Male sterility

There was an overall significant effect of hybrid genetic composition on all three measures of male sterility (χ^2^ association tests, Table 2). Male behavioral sterility was measured by observing male courtship behavior when confronted with a virgin female. Of the haploid pure species males 25% *N. vitripennis* and 62% *N. longicornis* males do not mate (*P* < 0.0001, Tables 2A,B). Most males that were unsuccessful either did not mount the female or did not progress to copulation after display. Hybrid haploid males have significantly higher levels of behavioral sterility; 80% of males with V cytotype and 92% of males with L cytotype do interrupt the mating process before or during copulation (effect of pure vs. hybrid composition, Table 2B). These high levels of behavioral sterility are mostly due to males that do not progress from display to copulation (see Koevoets et al., 2012a).

**Table 2A:** **Levels of sterility of haploid and diploid males from pure species and hybrid crosses**.

**ID**	**Cross type**	**Genetic composition**	**Ploidy**	**Behavioral sterility**		**Spermatogenic sterility**		**Overall sterility**	
				***N***	**% Sterile**	***N***	**% Sterile**	***N***	**% Sterile**
1	Control	100% V[V]	Haploid	49	24.5	35	0.0	49	24.5
2	Control	100% L[L]	Haploid	68	61.8	21	0.0	68	61.8
3	Hybrid	50%V/50%L[V]	Haploid	234	80.3	48	39.6	234	87.6
4	Hybrid	50%V/50%L[L]	Haploid	185	91.9	14	57.1	185	96.8
5	Control	100% V[V]	Diploid	25	16.0	18	5.6	25	32.0
6	Control	100% L[L]	Diploid	55	70.9	12	41.7	55	87.3
7	Hybrid	75%V/25%L[V]	Diploid	128	41.4	68	0.0	128	41.4
8	Hybrid	25%V/75%L[V]	Diploid	24	95.8	1	0.0	24	95.8
9	Hybrid	75%V/25%L[L]	Diploid	71	47.9	35	20.0	71	60.6
10	Hybrid	25%V/75%L[L]	Diploid	44	84.1	7	0.0	44	84.1
χ^2^ association test				*P* < 0.001		*P* < 0.001		*P* < 0.001	

*Hybrid classes (white cells) differed significantly for all three sterility measures, indicating an association between sterility and genetic composition (χ^2^ association tests, P < 0.001). V, vitripennis; L, longicornis; letter in square brackets indicates species cytotype*.

**Table 2B:** **Statistical comparisons of sterility levels**.

**ID**	**Cross type**	**Genetic composition**	**Ploidy**	**Behavioral sterility**	**Spermatogenic sterility**	**Overall sterility**
**EFFECT OF SPECIES IN HAPLOID RESP. DIPLOIDS**						
1	Control	100% V[V]	Haploid	^****^	ns	^****^
2	Control	100% L[L]	Haploid			
5	Control	100% V[V]	Diploid	^****^	^****^	
6	Control	100% L[L]	Diploid			
**EFFECT OF PLOIDY LEVEL IN PURE SPECIES WITH V, RESPECTIVELY, L CYTOTYPE**						
1	Control	100% V[V]	Haploid	ns	ns	ns
5	Control	100% V[V]	Diploid			
2	Control	100% L[L]	Haploid	ns	^**^	ns
6	Control	100% L[L]	Diploid			
**EFFECT OF PURE VS. HYBRID COMPOSITION IN HAPLOIDS WITH V, RESPECTIVELY, L CYTOTYPE**						
1	Control	100% V[V]	Haploid	^****^	^****^	^****^
3	Hybrid	50%V/50%L[V]	Haploid			
2	Control	100% L[L]	Haploid	^****^	^****^	^****^
4	Hybrid	50%V/50%L[L]	Haploid			
**EFFECT OF PURE VS. HYBRID COMPOSITION IN DIPLOIDS WITH V CYTOTYPE**						
5	Control	100% V[V]	Diploid	^*^	ns	ns
7	Hybrid	75%V/25%L[V]	Diploid			
5	Control	100% V[V]	Diploid	^****^	ns	^****^
8	Hybrid	25%V/75%L[V]	Diploid			
**EFFECT OF PURE VS. HYBRID COMPOSITION IN DIPLOIDS WITH L CYTOTYPE**						
6	Control	100% L[L]	Diploid	^*^	ns	^**^
9	Hybrid	75%V/25%L[L]	Diploid			
6	Control	100% L[L]	Diploid	ns	ns	ns
10	Hybrid	25%V/75%L[L]	Diploid			
**EFFECT OF CYTOTYPE IN HAPLOID, RESPECTIVELY, DIPLOID HYBRIDS**						
3	Hybrid	50%V/50%L[V]	Haploid	^***^	ns	^**^
4	Hybrid	50%V/50%L[L]	Haploid			
7	Hybrid	75%V/25%L[V]	Diploid	ns	^***^	^*^
9	Hybrid	75%V/25%L[L]	Diploid			
8	Hybrid	25%V/75%L[V]	Diploid	ns	ns	ns
10	Hybrid	25%V/75%L[L]	Diploid			
**EFFECT OF PLOIDY LEVEL IN HYBRIDS WITH V CYTOTYPE**						
3	Hybrid	50%V/50%L[V]	Haploid	^****^	^****^	^****^
7	Hybrid	75%V/25%L[V]	Diploid			
3	Hybrid	50%V/50%L[V]	Haploid	ns	ns	ns
8	Hybrid	25%V/75%L[V]	Diploid			
**EFFECT OF PLOIDY LEVEL IN HYBRIDS WITH L CYTOTYPE**						
4	Hybrid	50%V/50%L[L]	Haploid	^****^	^*^	^****^
9	Hybrid	75%V/25%L[L]	Diploid			
4	Hybrid	50%V/50%L[L]	Haploid	ns	^*^	^**^
10	Hybrid	25%V/75%L[L]	Diploid			
**EFFECT OF BACKCROSS IN HYBRIDS WITH V CYTOTYPE**						
7	Hybrid	75%V/25%L[V]	Diploid	^****^	ns	^**^
8	Hybrid	25%V/75%L[V]	Diploid			
9	Hybrid	75%V/25%L[L]	Diploid	^****^	ns	^*^
10	Hybrid	25%V/75%L[L]	Diploid			

*Statistical comparison of cross type, genetic composition and ploidy on sterility with Fisher exact or pairwise χ 2 (2 × 2) with Yates correction test; ns, not significant; p > 0.05*,

* *p < 0.05*;

** *p < 0.01*,

*** p < 0.001;

**** *p < 0.0001. After Bonferroni correction significance starts at p < 0.001*.

Pure species diploid males show 16% behavioral sterility in *N. vitripennis* and 71% in *N. longicornis* (*P* < 0.0001, Tables 2A,B), which is not different from haploid control males (effect of ploidy level in pure species, Table 2B). Comparison of control and hybrid diploid males is difficult because of the large difference in sterility of diploid males with V vs. L cytotypes. In general, diploid males with V cytoplasm have lower sterility than males with L cytoplasm. When comparing within cytotypes, diploid hybrid males with 75% L nuclear genome in V cytotype, show significantly higher levels of behavioral sterility than diploid control males. Comparison of haploid and diploid hybrid males shows an effect of the nuclear genome composition and cytotype. Diploid males with 75% V nuclear genome in V cytotype have 41% sterility compared to 80% in haploid 50%V [V] hybrids, and diploid males with 75% V nuclear genome in L cytotype have 48% sterility compared to 92% in haploid 50%V [L] hybrids. These diploid hybrid male values are significantly lower than haploid hybrids and diploid hybrids with 75% L genome in V and L cytotype (effect of ploidy level in hybrids, and effect of backcross in hybrids, Table 2B). These data show that being diploid rescues behavioral sterility to some degree, in particular when the fraction of vitripennis nuclear genome is high (75% vs. 25%).

Spermatogenic sterility of males was measured as the reduction in fertility; fertility being defined as the proportion of copulating individuals that did not produce daughters. Pure species haploid males (controls) of both *N. vitripennis* and *N. longicornis* have 0% sterility (Table 2). Haploid hybrids with a 50:50 V–L nuclear genome and V or L cytotype have 40%, respectively, 57% sterility which is significantly higher than controls (effect of pure vs. hybrid composition in haploids, Table 2B). Spermatogenic sterility of diploid control males is low in *N. vitripennis* (6%), but moderate in *N. longicornis* (42%) (not significant after Bonferroni correction, Table 2B). The reason for this difference in fertility between diploid males of the two species is not known. Three of the four groups of diploid hybrid males show full fertility whereas diploid males with 75% V genome in L cytotype (75% V[L]) have 20% spermatogenic failure. As most of the diploid hybrid males with 75% L genome were behaviorally sterile, sample sizes for these groups are low. As hybrid diploid males have lower sterility levels than hybrid haploid males, these results show that an increase in ploidy level from haploidy to diploidy rescues hybrid male spermatogenic sterility to a large extent.

Overall sterility rates are mostly determined by behavioral sterility and less so by spermatogenic failure. Diploid control males have similar levels of overall sterility as haploid control males. Haploid hybrid males and diploid hybrid males with 75% L nuclear genome have high sterility rates of 80–97% whereas diploid males with 75% V genome show intermediate levels of sterility. The largest increases in overall sterility are caused by being hybrid (effect of pure vs. hybrid composition), whereas increased ploidy level can reduce the effect (effect of ploidy level in hybrids, Table 2B).

#### Female sterility

There was an overall significant effect of hybrid genetic composition on all four measures of female sterility (χ^2^ association tests, Table 3). Female behavioral sterility was measured by observing mating behavior when confronted with a single male of one of the two species. Control *N. vitripennis* and *N. longicornis* females showed 5%, respectively, 21% unsuccessful matings when paired with their own species males (not significant after Bonferroni correction, Table 3B). Behavioral sterility levels of hybrid females are generally higher and vary from 16 to 82% (significant for 75% V in L cytotype, effect of pure vs. hybrid composition, Table 3B). Most females do not progress to copulation after being mounted (data not shown). There is a strong effect of test male species. Crosses where the test male matches the predominant nuclear composition of the hybrid female (e.g., *N. vitripennis* males with 75% V females) are more successful than those where the mismatch is larger. This effect is particularly strong for females with 75% V nuclear genome (effect of male species, Table 3B) and is likely the result of higher interspecific mate discrimination of *N. vitripennis* females toward *N. longicornis* males than vice versa.

**Table 3A:** **Levels of sterility of haploid and diploid females from pure species and hybrid crosses**.

**ID**	**Cross type**	**Genetic composition**	**Male species**	**Behavioral sterility**		**No fertilization**		**No offspring**		**Overall sterility**	
				***N***	**% Sterile**	***N***	**% Sterile**	***N***	**% Sterile**	***N***	**% Sterile**
1	Control	100% V[V]	V	55	5.45	50	0.0	54	1.9	55	9.1
2	Control	100% L[L]	L	57	21.1	41	4.9	57	15.8	57	31.6
3	Hybrid	75%V/25%L[V]	V	37	21.6	27	0.0	37	8.1	37	27.0
4	Hybrid	75%V/25%L[V]	L	21	61.9	8	0.0	21	4.8	21	61.9
5	Hybrid	25%V/75%L[V]	V	23	26.1	14	21.4	23	17.4	23	52.2
6	Hybrid	25%V/75%L[V]	L	31	16.1	22	9.1	31	9.7	31	35.5
7	Hybrid	75%V/25%L[L]	V	52	17.3	21	0.0	50	56.0	52	59.6
8	Hybrid	75%V/25%L[L]	L	39	82.1	5	0.0	35	65.7	39	87.2
9	Hybrid	25%V/75%L[L]	V	14	42.9	6	0.0	14	21.4	14	57.1
10	Hybrid	25%V/75%L[L]	L	14	28.6	7	0.0	14	21.4	14	50.0
χ^2^ association test				*P* < 0.001		*P* < 0.001		*P* < 0.05		*P* < 0.001	

*Overall sterility is the sum of behavioral sterility, fertilization failure and reduced fecundity (no offspring). Note that the different sterility components do not simply add up to overall sterility, because behaviorally sterile females may still produce offspring, i.e., all-male progenies as virgins. Hybrid classes (white cells) differ significantly for three of the four sterility measures, except “no fertilization,” indicating an association between sterility and genetic composition (χ^2^ association tests, P < 0.001). V, vitripennis; L, longicornis; letter in square brackets indicates species cytotype*.

**Table 3B:** **Statistical comparison of cross type, genetic composition and male species on four sterility measures with Fisher exact or pairwise χ^2^ (2 × 2) with Yates correction test**.

**ID**	**Cross type**	**Genetic composition**	**Male species**	**Behavioral sterility**	**No fertilization**	**No offspring**	**Overall sterility**
**EFFECT OF SPECIES**							
1	Control	100% V[V]	V	^*^	ns	ns	^**^
2	Control	100% L[L]	L				
**EFFECT OF MALE SPECIES**							
3	Hybrid	75%V/25%L[V]	V	^**^	ns	ns	^*^
4	Hybrid	75%V/25%L[V]	L				
5	Hybrid	25%V/75%L[V]	V	ns	ns	ns	ns
6	Hybrid	25%V/75%L[V]	L				
7	Hybrid	75%V/25%L[L]	V	^****^	ns	ns	^**^
8	Hybrid	75%V/25%L[L]	L				
9	Hybrid	25%V/75%L[L]	V	ns	ns	ns	ns
10	Hybrid	25%V/75%L[L]	L				
**EFFECT OF PURE VS. HYBRID COMPOSITION IN V CYTOPLASM WITH V MALE**							
1	Control	100% V[V]	V	^*^	ns	ns	^*^
3	Hybrid	75%V/25%L[V]	V				
1	Control	100% V[V]	V	^*^	ns	^*^	^****^
5	Hybrid	25%V/75%L[V]	V				
**EFFECT OF PURE VS. HYBRID COMPOSITION IN L CYTOPLASM WITH L MALE**							
2	Control	100% L[L]	L	^****^	ns	^****^	^****^
8	Hybrid	75%V/25%L[L]	L				
2	Control	100% L[L]	L	ns	ns	ns	ns
10	Hybrid	25%V/75%L[L]	L				
**EFFECT OF CYTOTYPE IN HYBRIDS**							
3	Hybrid	75%V/25%L[V]	V	ns	ns	^****^	^**^
7	Hybrid	75%V/25%L[L]	V				
4	Hybrid	75%V/25%L[V]	L	ns	ns	^****^	ns
8	Hybrid	75%V/25%L[L]	L				
5	Hybrid	25%V/75%L[V]	V	ns	ns	ns	ns
9	Hybrid	25%V/75%L[L]	V				
6	Hybrid	25%V/75%L[V]	L	ns	ns	ns	ns
9	Hybrid	25%V/75%L[L]	V				
**EFFECT OF HYBRID COMPOSITION IN V CYTOPLASM WITH V, RESPECTIVELY, L MALE**							
3	Hybrid	75%V/25%L[V]	V	ns	^*^	ns	ns
5	Hybrid	25%V/75%L[V]	V				
4	Hybrid	75%V/25%L[V]	L	^**^	ns	ns	ns
6	Hybrid	25%V/75%L[V]	L				
**EFFECT OF HYBRID COMPOSITION IN L CYTOPLASM WITH V, RESPECTIVELY, L MALE**							
7	Hybrid	75%V/25%L[L]	V	ns	ns	^*^	ns
9	Hybrid	25%V/75%L[L]	V				
8	Hybrid	75%V/25%L[L]	L	^***^	ns	^*^	^*^
10	Hybrid	25%V/75%L[L]	L				

*ns, not significant; p > 0.05*,

* *p < 0.05*,

** *p < 0.01*,

*** *p < 0.001*,

**** p < 0.0001. After Bonferroni correction significance starts at p < 0.001.

Sterility of females was further measured as the reduction in fertility; fertility being defined by two parameters, the proportion of individuals that produced daughters (fertilization) and the proportion of mated individuals that produced progeny following normal copulations. Note that individual brood sizes could not be compared between groups because the two parental species differ in average clutch sizes (higher in *N. vitripennis* than *N. longicornis*) and the number of surviving offspring is strongly affected by hybrid genotypes. All control *N. vitripennis* females fertilize eggs, i.e., have daughters among their progeny, and only 2% do not produce progeny (Table 3). Ninety-five percentage of control *N. longicornis* females fertilize eggs and 16% do not produce progeny, which is not different from *N. vitripennis* (effect of species, Table 3B) In most hybrid groups all females fertilize eggs, except for females with 75% longicornis nuclear genome that produce 21 and 9% progenies without daughters with *N. vitripennis* and *N. longicornis* male partners, respectively. The number of hybrid females that did not produce progeny varied from 5 to 66%, fecundity failure rates of 75% V[L] females being significantly higher than the pure species control (effect of pure vs. hybrid composition in L cytoplasm) and the hybrids with V cytotype (effect of cytotype, Table 3B). This is likely the result of strong post-zygotic incompatibilities in their haploid hybrid male offspring.

Overall female sterility rates were 9% for *N. vitripennis* and 32% for *N. longicornis*, (not significant after Bonferroni correction, effect of species, Table 3B) consistent with the general notion that *N. longicornis* females are somewhat harder to culture in the laboratory. Seven-five percent V[V] and 75% L[L] hybrid females do not differ in overall sterility compared to controls, but 75% L[V] and 75% V[L] females have significantly lower fecundity (effect of pure vs. hybrid composition in V, respectively, L cytoplasm, Table 3B), which again indicates an effect of mismatch between nuclear genome and cytoplasm. This effect is stronger for 75% vitripennis genome in L than V cytoplasm (effect of cytotype in hybrids, Table 3B). These results reiterate the strong post-zygotic incompatibility effects in haploid hybrid male offspring with L cytotype.

### Genotypic analysis

Transmission ratios of 32 microsatellites were tested for deviations from equality in the various experimental groups. Two microsatellites (Nv121 and Nv307) could not be reliably scored in all groups. Previous studies have shown various levels of cytonuclear incompatibilities between *Nasonia* species pairs, resulting in a higher recovery of maternal vs. paternal alleles in hybrids for some genomic regions (Niehuis et al., 2008; Koevoets et al., 2012a) whereas transmission ratios in pure species crosses are typically Mendelian. Table 4 shows for each hybrid group the transmission ratio distortion of the 32 microsatellites. Haploid hybrids with *N. vitripennis* cytotype did not show much distortion; one transmission ratio distorted locus (TRDL) on chromosome 1 (Nv311) comparable to previous results (Koevoets et al., 2012a,b) and a slight bias on chromosomes 2 and 5 that was significant in previous studies. Diploid hybrid males with 75% V genome and with 75% L genome in V cytotype exhibited no significant distortion, neither did diploid hybrid females with 75% L genome in V cytotype. Note that transmission ratios in hybrid females with 75% V genome in V cytotype were not investigated. These results mean that diploid VV and VL marker combinations in V cytotype have equal survival probability whereas haploid L alleles of Nv311 have reduced survival. In other words, diploidy rescues the negative cytonuclear interaction that is linked to the Nv311 marker, both for homozygous V and L alleles, indicating an effect of dosage.

**Table 4:**
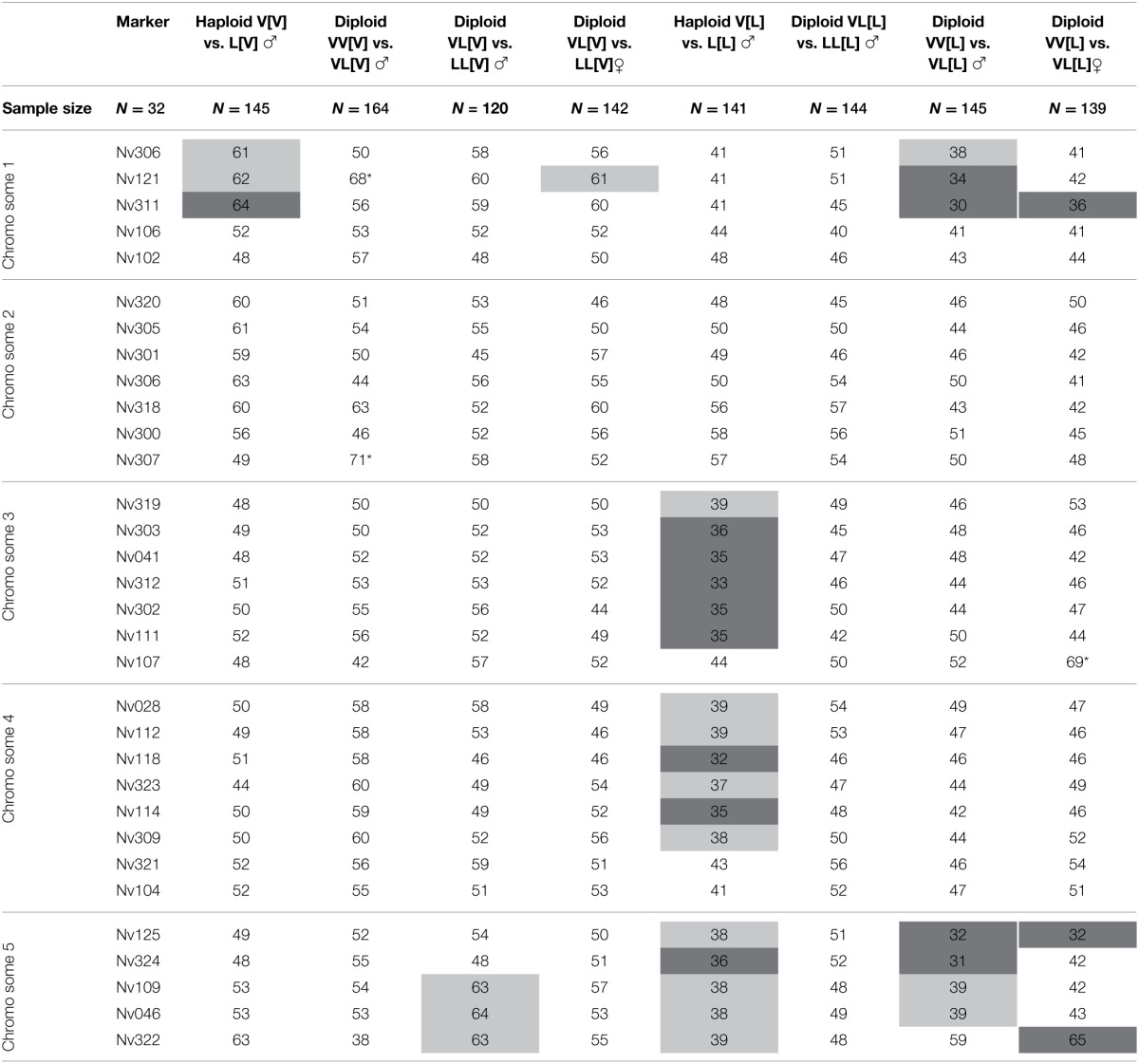
**Transmission ratio distortion in haploid and diploid males and females from hybrid crosses**.

*Each hybrid cross yielded two types of offspring genotypes for each of 32 microsatellite markers. Markers are distributed evenly over the five chromosomes and ordered according to their location. The numbers in each cell indicate the recovery rates of vitripennis alleles, in case of diploid individuals of the heterospecific complement only. The dark gray cells indicate markers with significant deviations from 50%, the light gray cells indicate markers with a recovery rate below 40% or above 60%, but that did not deviate significantly. The recovery rates indicated by an asterix were significantly distorted but this is likely caused by amplification problems rather than mortality*.

Haploid hybrid males with L cytotype exhibited significant transmission biases toward longicornis alleles on chromosomes 3, 4, and 5, comparable to previous results (Koevoets et al., 2012a,b). Diploid males with 75% L nuclear genome showed no distortions, i.e., neither LL nor LV genotypes had a survival advantage for any of the markers. In contrast, diploid males and females with 75% V genome showed biased recovery for a region on chromosome 1 and 5. Taken together, the loci on chromosome 1 reveal dosage effects of increased transmission distortion (two alleles cause more distortion than one), on chromosome 3 and 4 of dosage and dominance effect to recover hybrid inviability (a single V allele causes more distortion than either VV, VL, or LL allelic combinations), and on chromosome 5 of dominance of the longicornis allele to recover transmission distortion (LL and VL combinations yield equal transmission, but LV hybrids survive better than VV hybrids). Thus, diploidy rescues many of the negative cytonuclear incompatibilities, albeit in different ways.

Marker recovery rates were correlated between experimental hybrid groups to test whether regions with a specific effect in one class of hybrids had a similar effect in another class of hybrids. These correlations provided additional information to the χ^2^ analyses of biased recovery rates, as sequential Bonferroni corrections can filter out biased regions of potential interest. No significant correlations were found between the allelic recovery rates in diploid males and females with V cytotype. In contrast, significant correlation between diploid hybrid males and females with L cytotype indicated that similar genomic regions are involved in hybrid inviability between the two sexes (*R*^2^ = 0.161, *p* = 0.023; columns 9 and 10 in Table 4). Correlations of allelic recovery rates of haploid hybrid males with V and L cytotype were also significant (*R*^2^ = 0.175, *p* = 0.017; columns 3 and 7 in Table 4) although different in the two hybrid types (toward V in V cytotype and toward L in L cytotype). Allelic recovery rates in diploid males did not correlate for either cytotype. Correlations between haploid and diploid hybrids were only significant for males with L cytotype and the cross yielding VV[L] and VL[L] hybrids (*R*^2^ = 0.161, *p* = 0.023; columns 7 and 9 in Table 4). These results are generally consistent with the conclusions drawn from the individual TRDLs and point at a number of cytonuclear incompatibility loci whose effect on reduced viability is partially rescued by diploidy.

## Discussion

The aim of this study was to separately test the effect of sex and ploidy level on genomic incompatibilities in a species pair of *Nasonia* parasitoid wasps. We addressed whether haploid hybrid males of *N. vitripennis* and *N. longicornis* suffer more from incompatibilities due to their genotype (ploidy) or phenotype (sex). Males are normally haploid and females diploid in this haplodiploid hymenopteran. Crosses between *N. vitripennis* and *N. longicornis* yielded F_1_ hybrid females that were backcrossed to either pure species male to produce F_2_ haploid hybrid males and diploid hybrid females. Haploid F_2_ hybrid male offspring carried on average 50% nuclear genome of both parental species and a maternally inherited cytotype. Backcrossing reintroduced a complete (haploid) nuclear genome into the progeny, so that the maternal backcross has a full parental cytonuclear complement, whereas the parental backcross yields a mismatch between nuclear and cytoplasmic genomes. A subset of mated F_1_ females were treated with dsRNA against *transformer* to yield diploid offspring that developed as males. This resulted in groups of diploid hybrid males that could be compared with groups of diploid hybrid females with similar average genomic composition. Hybrid incompatibilities were measured as inviability and sterility in haploid and diploid males as well as diploid females to determine whether the incompatibilities in haploid male hybrids are due to haploidy or maleness. These comparisons enabled us to discriminate between faster-male and dominance effects, as faster-male effects would induce incompatibilities in both haploid and diploid males and dominance effects would rescue incompatibilities in diploid males and females, heterozygous for incompatibility alleles, but not in haploid males.

No significant differences in mortality rates were found between control (pure species) groups, indicating equal survival of haploid and diploid males and diploid females. Hybrid individuals had lower survival than pure species, but there was no clear effect of ploidy level or sex. Survival rates are based on the assumption of equal fertilization proportions of injected and uninjected females, as diploid male and female individuals are always accompanied by haploid males in their broods. We have no indications that RNAi knockdown of *transformer* affects the fertilization proportion of females (unpublished results).

Despite the small effects on hybrid inviability, the genetic analyses of surviving hybrids show some clear signs of transmission ratio distortion. Hybrids with V cytotype have, except for one locus on chromosome 1 in haploids, allelic recovery rates that do not differ from equality. In hybrids with L cytotype, comparisons between groups showed several genomic regions, i.e., on chromosomes 1, 3, 4, and 5, where L alleles were recovered at higher rates than V alleles. This can only be explained by cytonuclear incompatibility, where L alleles survive better in L cytotype, and indicate that L cytotype is more restrictive in interaction with V alleles, consistent with previous studies (Beukeboom and van den Assem, 2001).

Distortions on chromosome 1 point toward a dosage effect, on chromosome 3 and 4 toward dosage and dominance, and on chromosome 5 toward dominance. A dosage effect means that two copies of a nuclear genome of either species has a differential effect on hybrid survival. A dominance effect means that a single copy of the L genome in L cytotype can rescue the incompatibility.

Dosage effects are not supportive for either dominance or faster-male effects. Dominance predicts a difference in survival of homozygotes vs. heterozygotes, whereas faster-male effects predict a lower survival of diploid males than females. Furthermore, the genomic region on chromosome 1 proved more deleterious in diploid homozygotes than haploid hemizygotes. These results are suggestive of a dosage effect that aggravates incompatibility. Thus, carrying incompatible gene-combinations in a higher dose can both positively and negatively affect the manifestation of hybrid incompatibilities. In relation to the suggested disruption of the oxidative phosphorylation pathway in *Nasonia* (Ellison et al., 2008; Niehuis et al., 2008) and other (Barreto and Burton, 2013) hybrids it is possible that incompatible gene-combinations lead to a lowered ATP production. Having a double amount of partial dysfunctional proteins might induce the production of sufficient ATP compared to having a single amount, explaining the less severe effect in diploid homozygotes. Conversely, the level of a malignant product may only become lethal at a diploid dose compared to a haploid dose. Consistent with this interpretation, many hybrid cytonuclear incompatibilities are mapped to tRNA and rRNA in both mitochondrial and nuclear genomes (Burton and Barreto, 2012; Burton et al., 2006; Meiklejohn et al., 2013), suggesting that protein production regulation (translation and transcription) play a major role in cytonuclear co-evolution.

The hybrid sterility experiments showed that the nuclear compositions of hybrids and their mates are important determinants of the success of mating interactions. The results are consistent with those of Clark et al. (2010) on *N. vitripennis–N. giraulti* hybrids that showed high levels of behavioral sterility in one cross direction only (with the giraulti cytotype). Asymmetry in hybrid dysgenesis between reciprocal crosses is commonly observed and suggest an effect of genetic elements that are uniparentally inherited, such as X-chromosomes and mitochondria (Turelli and Moyle, 2007; Clancy et al., 2011). In our study, increasing the ploidy level had a positive effect on the course of male courtship, but the percentage *N. vitripennis* alleles was more indicative of the success of courtship as a whole. This suggests that being diploid positively affects the general fitness of males, but that the type of courtship that is displayed is greatly affected by compatible alleles and not ploidy *per se*. In line with our results, Ellison and Burton (2006) also found that having a full parental cytonuclear complement increased the fitness of hybrids of the copepod *Tigriopus californicus*. A potentially complicated factor in quantifying behavioral sterility in *Nasonia* are differences in pre-zygotic isolation between the species. *N. longicornis* females discriminate stronger against *N. vitripennis* males than *N. vitripennis* females do against *N. longicornis* males. We therefore chose to only use *N. vitripennis* females for measuring the behavioral sterility.

Spermatogenic sterility of males was quantified by absence of females among their offspring following successful copulation. Haploid hybrid males showed high levels of spermatogenic failure due to the inability to produce sperm, to transfer sperm and/or the production of sperm that cannot be stored/used by the female. Clark et al. (2010) observed lower sperm counts rather than non-functional sperm in hybrid males of *N. vitripennis* and *N. giraulti*. Diploid hybrid males were rescued from spermatogenic failure, regardless of their nuclear composition. Most hybrid female groups showed no abnormalities in offspring production. The exception is the lowered fecundity of 75% V[L] females. This is most likely due to F_3_ hybrid inviability rather than F_2_ sterility and illustrates the difficulty to distinguish these two effects for females.

The components of physiological sterility differ between males and females and thus concern different biological processes and likely underlying genes. Notwithstanding this notion, it is obvious that hybrid diploid males and females are largely fertile, compared to the high frequencies of spermatogenic failure in haploid hybrid males. This points at dominance effects on cytonuclear incompatibilities, as faster-male effects predict comparable sterility of haploid and diploid males. A molecular analysis of the genetic basis of hybrid sterility has however not been performed and might reveal alternative mechanisms like dosage, as in the molecular analysis of inviability (see below). Once the genomic regions responsible for hybrid spermatogenic failure are identified, their effect in homozygotes and heterozygotes can be compared to further disentangle the genetic basis of hybrid sterility in *Nasonia*.

The overall sterility of individuals is determined by different factors and many biological pathways may be involved. As these pathways differ between the types of sterility and between males and females, comparing sterility between males and females is challenging. However, when comparing the overall sterility of males and females one can get information about their ability to reproduce, which is important in the light of reproductive isolation between species. Hybrid male overall sterility tended to be higher than hybrid female overall sterility, although only significantly for hybrids with 75% V nuclear genome in L background. This shows that diploid hybrid males might be slightly more susceptible to sterility than diploid hybrid females, which would be in line with the fact that males are normally haploid and lack epistatic interactions between gene copies. In conclusion, haploid male sterility can be predominantly ascribed to dominance effects rather than faster-male effects, as under faster-male effects diploid males are predicted to have higher levels of sterility and diploid hybrid females are predicted to have none.

### Haldane's rule in nasonia

A large proportion of haploid hybrid males showed behavioral sterility. Many individuals seem to suffer from morphological and physiological defects, have decreased locomotor activity, and are too weak to perform courtship behavior, consistent with previous reports (Beukeboom and van den Assem, 2001; Bordenstein et al., 2001; Clark et al., 2010). Our data showed that behavioral abnormalities in hybrid males are largely determined by their nuclear composition in combination with the cytotype. Together with the large impact of female receptiveness to male courtship, this poses limits to the use of courtship behavior for studying the genetic mechanisms of sterility underlying Haldane's rule. Spermatogenic failure, on the other hand, although indirectly measured, was clearly rescued by diploidy in hybrid males. Sperm mobility in haploid hybrids may be more affected than in diploid hybrids by reduced ATP production as a consequence of a mismatch between nuclear and mitochondrial genes. Spermatogenic failure seems to support the dominance theory, as faster-male effects would have induced more sterility in diploid males as well. This shows that recessive negative epistatic interactions are likely the cause for the high levels of sterility in *Nasonia* haploid hybrid males. The lack of evidence for faster-male effects is consistent with the idea that sexual selection is predicted to be less effective in haplodiploid systems due to lack of heterologous sex-chromosomes (Reeve and Pfennig, 2003).

Survival analysis showed comparable mortality rates between diploid males and females. Furthermore, genetic analysis of hybrid offspring pointed at two independent mechanisms rescuing hybrid inviability: dominance and dosage. In some hybrids, particularly those with longicornis cytotype, presence of a single L allele reduced hybrid inviability, whereas in other hybrids presence of two heterospecific alleles increased or decreased hybrid inviability compared to one heterospecific allele (hemizygotes or heterozygotes). This supports the claim that Haldane's rule is a composite phenomenon that cannot be explained by a single mechanism (Coyne, 1992).

The role of dosage, although ignored in most studies, is not novel in explaining Haldane's rule. Orr (1993) studied inviability in *Drosophila* hybrids and found that females with heterozygous X-chromosomes were viable, whereas females homozygous for the incompatible (non-congener) X-chromosome were inviable, like males with a single X-chromosome, and thus dosage did not rescue female inviability. Turelli and Orr (1995) and Orr and Turelli (1996) considered the cumulative additive effect of BDM incompatibilities more formally. In contrast to studies on diploids that consider the disruption of the ratio of sex chromosomes to autosomes, our study considers the ratio between ploidy level (haploidy vs. diploidy) and cytoplasmic genes. In *Nasonia* hybrids, we observed a clear interaction between ploidy level and cytoplasmic genotype, with evidence for both dominance and dosage effects of hybrid inviability loci. The unique advantage of this haplodiploid system is that it allows for easy detection of such cytonuclear interactions. Dosage effects might have remained unidentified in BDM studies in diploid species due to limitations of the sex determination system: no genomically identical males and females can be created.

We have previously shown that temperature affects the strength at which negative epistatic interactions are expressed in *Nasonia* (Koevoets et al., 2012b; see also Hoekstra et al., 2013 for a *Drosophila* example). A follow up experiment could measure to what degree the different rescue mechanisms, such as dominance and dosage, are influenced by elevated temperature. Another factor that may play a role in the severity at which hybrid incompatibilities are manifested is the microbiome. Bruckner and Bordenstein (2013) reported that survival of *Nasonia* hybrids increased when the hybrids were cultured in the absence of species-specific gut bacteria. Some of our measured dominance and dosage effects in hybrids may therefore be influenced by altered interactions between nuclear gene products and microbes. This could be tested by performing similar hybridization experiments under microbe-free conditions.

One unique aspect of our study is the availability of diploid hybrid males that are genomically identical to hybrid females. This allowed us to for the first time compare diploid F_1_ males and females with one full genomic complement of two parental species, and to separate the effects of ploidy level and sex on hybrid incompatibilities. Diploid males and females were rather similar for the fitness parameters measured and showed fewer incompatibilities than haploid hybrid males. We therefore conclude that higher incompatibilities in *Nasonia* males are mostly due to ploidy level rather than sex. This is the second study using sex-reversal to examine the genetic mechanisms underlying hybrid incompatibility. Malone and Michalak (2008) used hormonal treatment and implantation to revert sexual development of hybrid *Xenopus* frog tadpoles. In these normally female heterogametic frogs, male hybrids are sterile and female hybrids are fertile, which led the authors to conclude that faster-male effects were responsible for the observed hybrid sterility as both normal and sex-reversed males were sterile. Faster-male effects under female heterogamety are the basis of most exceptions to Haldane's rule (Schilthuizen et al., 2011). One important drawback of the *Xenopus* study is that tadpole sex-reversal occurred after the onset of embryogenesis. At this time, sex-specific gene-expression is likely to have irreversible started a range of sex-specific developmental processes. The sex-reversal as exploited in our study has the advantage that female development is prevented all the way, as the parental RNAi procedure directs diploid eggs toward male development before embryogenesis starts.

Summarizing, hybrid fertility appears to be stronger affected by hybridization than hybrid viability, which is consistent with previous findings in insects (Wu and Davis, 1993; Coyne and Orr, 1998). By comparing hybrid incompatibilities of haploid males to diploid males and females we were able to show rescue effects of diploidy on both inviability and sterility, indicative of dominance effects. Molecular analyses showed that not only dominance effects rescue inviability, but that also dosage plays a role in the severity at which BDM incompatibilities are expressed. This role of dosage can be positive or negative, and likely depends on the nature of the underlying genes, which awaits future study. Our study further points toward an important role of cytonuclear rather than nucleo–nucleo interactions in causing post-zygotic incompatibilities. To what extent this is a specific feature of the *Nasonia* system, or possibly of haplodiploid systems, remains to be seen. Of interest, Clancy et al. (2011) state that cytoplasmic male sterility in animals may be more common than previously thought and may be hard to uncover in diploid laboratory strains. Although *Nasonia* is a haplodiploid organism that lacks heteromorphic sex chromosomes, our study of hybrids in this system has shown that it can be informative about the processes that underlie Haldane's rule, a notion that Haldane himself already made in his original description of the phenomenon.

### Conflict of interest statement

The authors declare that the research was conducted in the absence of any commercial or financial relationships that could be construed as a potential conflict of interest.
